# The Fluorescent Cell Line SW620-GFP Is a Valuable Model to Monitor Magnetic Hyperthermia

**DOI:** 10.3390/bioengineering11070638

**Published:** 2024-06-21

**Authors:** Saray Rosales, Rodolfo Hernández-Gutiérrez, Alma Oaxaca, Zaira López, Norberto Casillas, Peter Knauth, Luis H. Quintero, José A. Paz, Francisco Cholico, Celso Velásquez, Mario E. Cano

**Affiliations:** 1 Centro Universitario de Ciencias Exactas e Ingenierías, Universidad de Guadalajara, Marcelino García Barragan 1421, Guadalajara 44430, Jalisco, Mexico; saray.rosales@uteg.edu.mx (S.R.); norberto.casillas@academicos.udg.mx (N.C.); 2 Centro de Investigación y Asistencia en Tecnología y Diseño del Estado de Jalisco A.C., Av. Normalistas 800, Guadalajara 44270, Jalisco, Mexico; almave15@hotmail.com; 3 Centro Universitario de la Ciénega, Universidad de Guadalajara, Avenida Universidad 1115, Ocotlan 47810, Jalisco, Mexico; zaira.lopez@academicos.udg.mx (Z.L.); peter.knauth@academicos.udg.mx (P.K.); jose.apaz@academicos.udg.mx (J.A.P.); apolinar.cholico@alumnos.udg.mx (F.C.); 4 Centro Universitario de Ciencias Económico Administrativas, Universidad de Guadalajara, Periférico Norte 799, Col. Los Belenes, Zapopan 45100, Jalisco, Mexico; hector.quintero@academicos.udg.mx; 5 Centro Universitario de los Valles, Universidad de Guadalajara, Carretera Guadalajara—Ameca Km. 45.5, Ameca 46600, Jalisco, Mexico; celso.velasquez@valles.udg.mx

**Keywords:** fluorescence, nanoparticles, hyperthermia

## Abstract

In this work, the cell line SW620-GFP has been used in a complete magnetic hyperthermia assay, from the preparation of the ferrofluid with folate-coated iron oxide nanoparticles to in vivo experiments. The physical and chemical characterization of the nanoparticles evidenced their superparamagnetic behaviour, an average diameter of 12 ± 4 nm, a 2 nm coat thickness, and a high-power loss density. The main innovation of the work is the exclusive capability of viable SW620-GFP cells to emit fluorescence, enabling fast analysis of both, cell viability in vitro with an epifluorescence microscope and tumour size and shape in vivo in a non-invasive manner using the iBox technology. Moreover, with this imaging technique, it was possible to demonstrate the successful tumour size reduction in mice applying magnetic hyperthermia three times a week over 3 weeks.

## 1. Introduction

The importance of magnetic hyperthermia (MHT) as a complementary oncologic treatment has been demonstrated in recent experiments conducted on patients with prostate cancer under an Investigational Device Exemption (IDE) [1]. It is expected that focal ablation, induced by heating the prostate tumour, will result in an increased survival rate for the patients. Several decades of experience were necessary to improve the synthesis of the nanomaterials and their coatings to increase biocompatibility. Moreover, many in vitro and in vivo experiments were needed to reach this FDA approval. Nevertheless, it is essential to continually criticize MH in order to elucidate to clarify its current potential and limitations [2]. Some authors stressed the need to standardize the synthesis and characterization of the ferrofluids, to improve reproducibility among independent experiments [3]. Although magnetite ferrofluids have shown to be good candidates for MH and despite the latest advances, the tumour-selectivity still should be improved [4]. However, the benefits of MH to increase the efficacy of cancer treatment when combined with conventional therapies are evident [1,5].

For over 20 years, one of the most commonly used ferrofluids in MH [6] or other biomedical applications, like magnetically targeted drug delivery [7,8,9] or MRI [10,11], has been a colloidal suspension of iron oxide nanoparticles coated with folic acid or organic polymers, such as polyethylene glycol [12] or polyethylenimine [13]. These ferrofluids have gained popularity, due to the affinity of folic acid to the folate receptors, which are over-expressed in many cancer cells [14].

In order to ensure good colloidal stability of the magnetic nanoparticles, the ferrofluids used in MH have adequate physical–chemical properties: Preferably, they should be superparamagnetic iron oxide nanoparticles (SPIONs) with diameters ranging from 10 to 20 nm and associated with high specific absorption rate (SAR) of several tens of W/g. Additionally, in numerous in vitro experiments, and using different cell lines, it has been observed that an organic coating of the SPIONs enhances their cellular uptake and reduces their cytotoxic effects [15,16,17,18]. Cell viability is frequently determined by using a tetrazolium dye (e.g., MTT) or a direct vital staining method, such as trypan blue. After standardization of a technique to quantify cytotoxicity, a differential analysis of the cell viability between MH-treated cells and the corresponding control samples must be able to demonstrate the efficacy of the ferrofluid and provide a starting point to investigate the cell death mechanisms.

A ferrofluid with suitable physical and chemical properties, low cytotoxicity, and high efficacy in vitro for MH becomes a strong candidate for in vivo trials with rodent specimens [19,20]. It is crucial to monitor the evolution of the tumour and the survival rate of the animals in these trials. Usually, callipers are used in these types of assays to continually measure the diameter of cancerous tissue and to estimate its corresponding volume, assuming spherical or ellipsoidal shapes [21,22].

The main objective of this work is to propose a versatile visual alternative to carry out in vitro and in vivo experiments on MH. This can be achieved by using the fluorescent cell line SW620-GFP to determine cell viability in vitro using epifluorescence microscopy and to measure the tumour volume non-invasively in vivo using the iBox fluorescence imaging technology. These procedures will be compared to the results of other cell viability tests, such as the traditional WST-1 and other vital staining techniques, as well as the tumour volume estimations using callipers.

## 2. Materials and Methods

### 2.1. Synthesis of SPIONs

The ferrofluid with magnetite nanoparticles was prepared using the coprecipitation method with FeCl_3_ and FeSO_4_ × 7H_2_O as precursor salts and NH_4_OH as the precipitating agent as described in [20,23]. The nanoparticles were coated by adding 126 mg folate (Sigma-Aldrich, 97% purity, Milwaukee, WI, USA) to 100 mL ferrofluid (4 mg/mL), which then was sonicated (30 kHz at 39 °C) for 45 min. Afterwards, the solution was stirred at 250 rpm for 24 h, then the SPIONs were washed (and centrifuged at 10,000 rpm) three times, and finally re-suspended in deionized water with a final concentration of 10 mg/mL.

### 2.2. Physical and Chemical Characterization of the SPIONs

The X-ray diffractometer (XRD) Empyrean (Malvern Panalytical, Malvern, UK) was used to analyse the crystal structure of the SPIONs. The interval of 5° ≤ 2θ ≤ 80° was covered by applying uniform steps of Δθ = 0.02° and a sampling time of t = 30 s. Three samples of SPIONs, dried at room temperature, were measured.

To determine the shape and statistical size distribution of the SPIONs, several TEM micrographs were captured using a JEM-2100 system (JEOL, Tokyo, Japan). Aliquots of SPION samples (1:100 diluted) were deposited and dried on the corresponding FCF-200-Cu sample holders (grids).

FTIR spectra of three samples were compared to analyse the union between magnetite and folate: (i) pure magnetite nanoparticles (5 mg); (ii) pure folic (10 mg) acid; and (iii) dried SPIONs (10 mg). A Thermo Scientific spectrometer (Nicolet iS5, Waltham, MA, USA) was used in attenuated total reflection modality (ATR) to register the absorbance over the interval of 4000 < ν < 400 cm^−1^.

The content of folate on the SPIONs, i.e., linked with the magnetite, was determined using a Thermogravimetric Analyzer (TGA) (ISI 1000, Twin Lakes, WI, USA) by evaporating the organic material. Therefore, three samples of approximately 10 mg of SPIONs were heated in an inert N_2_ atmosphere at a heating rate of 10 K/min within the temperature range of 35 ≤ T ≤ 800 °C. The relative mass dependence on the temperature was continuously recorded and compared to the control, i.e., dried magnetite nanoparticles.

A VersaLab vibrating sample magnetometer (VSM) (Quantum Design, San Diego, CA, USA) was used to magnetize dried samples of SPIONs and magnetite nanoparticles, within the interval of magnetic fields H from −30 kOe to 30 kOe, in order to determine their magnetic behaviour. Samples of 5 mg were deposited in diamagnetic holders and the induced magnetization M was measured at room temperature by recording hysteresis loops M vs. H. Additionally, the Zero Field Cooling—Field Cooling (ZFC-FC) protocol was followed to determine M by applying H = 100 Oe over a temperature range of 50 < T < 400 K.

The colloidal stability of the SPIONs was analysed by measuring their Z potential, using a Zetasizer (Nano ZS90, Malvern, UK) with the capillary cell DTS1070. The pH of the SPION samples was modified within a range of 2 < pH < 11 by adding acid (HCl) or alkaline (NaOH), and the resulting Zeta-potentials were measured.

The heating capability of the SPIONs in alternating magnetic fields was determined using a previously reported [24] magnetic-calorimeter system (MX Pats. 65,340, Mx/a/2018/002848, Guadalajara, Jalisco, Mexico), which can be operated in a frequency range of 185 kHz < f < 530 kHz and applies a magnetic field of 0 < H < 366 Oe. The temperature of the sample was measured with a fluoroptical sensor Luxtron-One. Three 1 mL samples of SPIONs, diluted to a concentration of 1 mg/mL, were pipetted into 2 mL Eppendorf tubes. Then, each sample was placed in the cavity of the external solenoid of the induction heater to be irradiated. After the alternating magnetic field was induced, the temperature of the samples was measured for 60 s. As a control, 1 mL of distilled water was irradiated under the same conditions to estimate the background noise of the measurements.

### 2.3. Cell Line, Growing Conditions, and Relative Cell Viability (RCV)

The human colon cell line SW620-GFP (#CCL227, ATCC, Manassas, VA, USA), which stably expresses the green fluorescent protein (GFP), was cultured in RPMI1640 (ATCC) supplemented with 10% FBS (Biowest, Kansas City, MO, USA) at 37 °C, with 4% CO_2_ and 95% relative humidity. Two-millilitre glass wells containing a cover slide were inoculated with 1.5 × 10^5^ cells/mL and incubated for 24 h to promote their attachment. After reaching 80% confluence, SPIONs at different concentrations were added into each well, and the cells were incubated for a further 24 h. To quantify changes in the metabolic activity of the cells, the medium was removed, the cells were washed three times with phosphate-buffered saline (PBS), and 1 mL of fresh medium containing 20 μL WST-1, a water-soluble tetrazolium (Takara Bio, Mountain View, CA, USA), was added. After 4 h of incubation, the medium was centrifuged (1 min, 10,000 rpm; Eppendorf 5415 D, Hamburg, Germany), and the absorbance of the supernatants was measured at 440 nm using a spectrophotometer (Mecasys Biopop, Daejeon, Republic of Korea). The measured absorbance is proportional to the metabolic activity of the cells and can be expressed as relative cell viability (RCV) when compared to the negative control.

As an independent complement to the WST-1 test, cell viability was estimated by Trypan blue staining. The remaining cells, which were grown on cover slides, were incubated with 500 μL growth medium and 10 μL Trypan blue (0.05%, Biowest) for 5–10 min at room temperature. Subsequently, the cover slides were washed with PBS and observed under a microscope (Axioskop 40FL, Zeiss, Oberkochen, Germany). Additionally, the RCV was quantified by estimating the confluence of green fluorescing cells using an epifluorescence microscope (Axioskop 40FL).

### 2.4. Cytotoxicity and In Vitro Assays of Magnetic Hyperthermia

When the cells, incubated as described in Section 2.3, reached 80% confluence, the ferrofluid of SPIONs was added at a final concentration of 2 mg/mL. The growth control contained only cells, while the mock control contained SPIONs but was kept at 37 °C. All other wells were irradiated using the induction heater for 20 min (530 kHz) with different amplitudes, allowing temperatures T = 39, 41, 43, 45, and 48 °C (±0.5 °C). Afterwards, the cells were incubated for 24 h under standard conditions prior to determining their RCV (see Section 2.3). The experiment was repeated four times to estimate the correlation between the average RCV and T.

### 2.5. In Vivo Assays of Magnetic Hyperthermia

Nine female nu/nu mice, 4- to 5-week-old (Bioterio de Morelos, Tlayacapan, Mexico), were used for the in vivo studies. According to the Helsinki Declaration (1964), the animals were housed in groups of three animals in filter-capped polycarbonate cages. The cages were kept in a ventilated room with controlled temperature (24 ± 1 °C) and humidity (50 ± 10%) and maintained on a 12 h/12 h light/dark cycle. After 2 weeks of acclimatization to the laboratory, all animals received a subcutaneous injection with SW620-GFP cells to generate and grow tumours for a fortnight. Then, the mice were divided into three groups: In group 1 (n = 4), the mice were treated with an intratumoural injection of the ferrofluid of SPIONs plus MH. The mice in group 2 (n = 3) were treated with an intratumoural injection of ferrofluid of SPIONs but without MH. Group 3 (n = 2) served as the negative control; thus, the mice were neither injected nor irradiated. Finally, the tumour development was monitored for 3 weeks, and the study concluded with the euthanization of the animals.

To induce a subcutaneous tumour, 6 × 10^6^ cells in 50 μL RPMI-1640 medium were injected into the back of the rodents, in such a manner, that the mice could not reach this area with their limbs. The tumour development was continually monitored; once the tumour diameter reached approximately 1.5 cm, mice of groups 1 and 2 were anaesthetized with 25% isoflurane in room air and 50 μL of ferrofluid with a concentration of 10 mg/mL of SPIONs were injected by direct puncture. The mice in group 1 were exposed to a magnetic field with a frequency of 190 kHz and an amplitude of 50 mT for 40 min three times a week.

To determine tumour sizes, two alternative methods were used: (a) volumetric measurement using a micrometric calliper (with 10 μm resolution) to determine the length (L) and width (W) of the tumour and (b) the iBox explorer image microscope. Both methods use the formula *V* = 0.5 × *L × W*^2^ [21] to estimate the tumour volume. The second method is an excellent option because the iBox is able to generate colour maps according to the intensity of the fluorescence emitted by the viable SW620-GFP cells, similar to a “radiography” of the tumour. After 21 days, the mice were sacrificed via cervical dislocation, and the tumours were removed to determine their precise volume by weighing the liquid displaced from a glass filled with water.

## 3. Results and Discussion

### 3.1. Physical and Chemical Characterization

**Figure 1:**
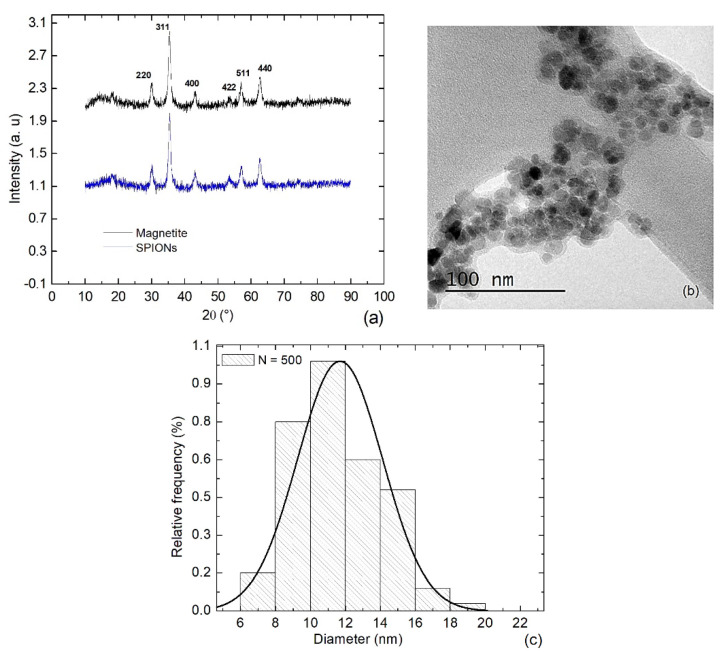
(**a**) XRD spectra of the magnetite nanoparticles (black line) and SPIONs (blue line); (**b**) TEM micrograph of the SPIONs; and (**c**) the statistics on size distribution.

According to the XRD spectra (Figure 1a), both the dried magnetite nanoparticles and the SPIONs exhibited the expected magnetite structure. The Miller indices (2 2 0), (3 1 1), (4 0 0), (4 2 2), (5 1 1), and (4 4 0) were present in both samples, with the typical main peak at 35.7° (in 2θ units) as published in the file No. 19-0629 of JCPDS. The spherical shape of the SPIONs was documented by TEM (Figure 1b), where also a slight polydispersity in the size was observed (like [25]). The statistical analysis of size indicates a Gaussian distribution with an average diameter of σ = 12 ± 4 nm (Figure 1c).

**Figure 2:**
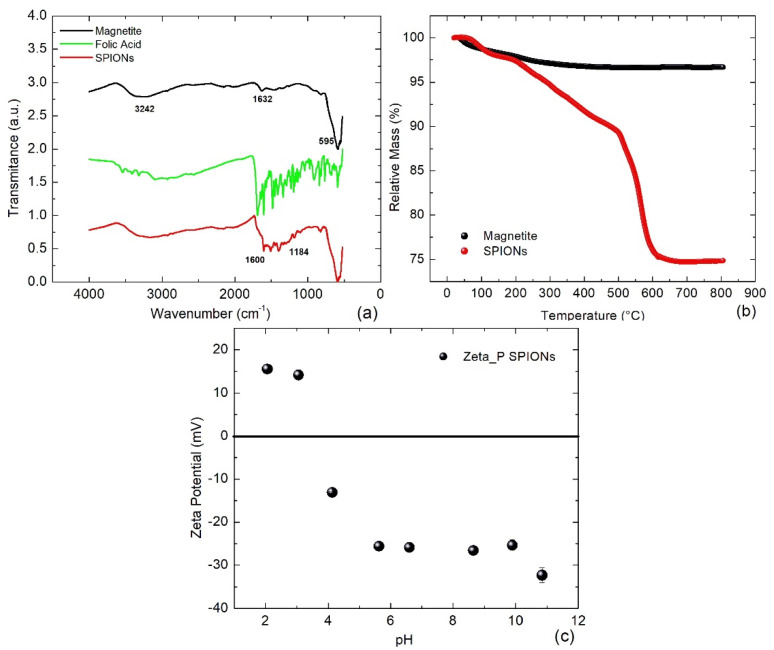
(**a**) FTIR spectra of the magnetite (black line), folate (green line), and SPIONs (red line); (**b**) TGA analysis of magnetite (black line) and SPIONs (red line); and (**c**) Zeta potential of the ferrofluid with SPIONs.

A comparison of the FTIR spectra indicates the successful coating of magnetite with folate as shown in Figure 2a: The transmittance signal of the SPIONs (red line) exhibits the typical resonant vibrations of magnetite (black line) near the wave numbers 3240, 1632, and 595 cm^−1^, which has been affected by the vibrations of folate (green line) at wave numbers 3550–3320 cm^−1^ and 1693–500 cm^−1^.

According to TGA measurements, the magnetite nanoparticles lost about 3% of their mass between 100 and 400 °C (Figure 2b, black line), indicating a low surface water content. On the other hand, SPIONs lost approximately 3% of their mass at temperatures between 100 and 200 °C, which corresponds to loosely bound surface water and was followed by a further mass reduction of about 7% up to 540 °C, which represents the degradation of the functional groups of the folate. Between 540 and 600 °C, the SPIONs lost an additional 15% of their mass associated with the decomposition of pyrolysis products formed in the previous step [26]; then, the mass remained constant until 800 °C. Therefore, the fraction of evaporated mass *η_FA_* = 0.22 corresponds to folate (i.e., *η_M_* = 0.75 is the fraction of magnetite). Assuming a density of *ρ_FA_* = 1.6 g/cm^3^ for folate and of *ρ_M_* = 5.3 g/cm^3^ for magnetite, then the thickness of the folate coat can be estimated to be Δ = 2.1 nm, when applying the formula published in [27]:

$$\Delta =\frac{\sigma }{2}\left[{\left(\frac{\eta_{M}\rho_{FA}}{\eta_{M}\rho_{FA}+\eta_{FA}\rho_{M}}\right)}^{-1/3}-1\right]$$

The Zeta potential measurements revealed that the SPIONs have a negative charge at a pH > 3.5 and exhibited good colloidal stability within the range of pH 5–11 (Figure 2c). This is an important property because the negative charge of the SPIONs promotes their endocytosis and, at the same time, the ferrofluid maintains colloidal stability in biological experiments, where the pH of the system has to be very close to pH = 7.4. A measurement of the polydispersity index PDI when pH ≈ 6.8 reaches PDI ≈ 0.22, which is a relatively low homogeneity (according to the size distribution of Figure 1c), but it is considered sufficient for magneto-calorimeter tests and magnetic hyperthermia applications [20,23,28,29].

**Figure 3:**
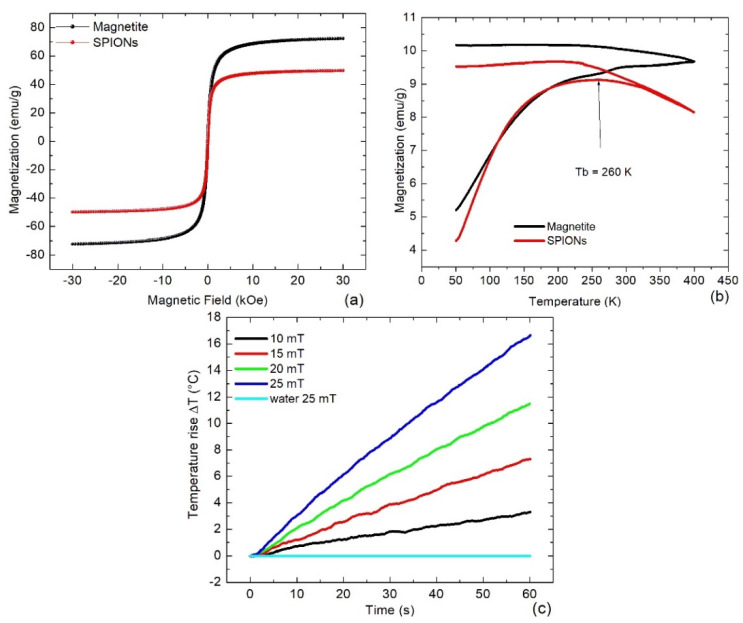
(**a**) VSM measurements of magnetite (black line) and SPIONs (red line); (**b**) their corresponding ZFC-FC traces; and (**c**) calorimetric measurements of the ferrofluid with SPIONs irradiating a magnetic field of 530 kHz frequency and amplitudes from 10 to 25 mT.

The magnetic saturation of the SPIONs (red line) was 50 emu/g approximately 30% lower than the one of pure magnetite (black line) (Figure 3a). This reduction was attributed to the diamagnetic folate and water that covered the surface of the SPIONs. A deep analysis of the hysteresis curve revealed for SPIONs and pure magnetite a coercivity of 2 Oe and 5 Oe, respectively. These small values suggested a superparamagnetic behaviour for both types of samples. However, the magnetization measured following the ZFC-FC protocol, showed that only the SPIONs had a deflection point in the ZFC curve at the blocking temperature of T_b_ = 260 K (Figure 3b, red line). Therefore, only the SPIONs exhibited superparamagnetic properties, which can be attributed to the coating with folate.

On the other hand, the heating capacity of the SPIONs at a concentration of 1 mg/mL strongly depended on the intensity of the alternating magnetic field (Figure 3c). First of all, when water was irradiated, and even at the highest intensity of 25 mT, only a non-significant temperature increase was observed. In contrast, within only 1 min samples containing SPIONs could be heated up by 3.5 °C at 10 mT and by 17 °C at 25 mT. Therefore, the heating rate achieved using these types of SPIONs seems to be adequate to carry out MH. For this purpose, it should be considered that in vitro cells can grow in 1 mL of liquid medium, but in vivo, tumours of 1 cm^3^ are typically induced in mice for biomedical experiments. Additionally, an increase in temperature of 8 °C is sufficient to induce irreversible cell damage.

### 3.2. Results of Cytotoxicity and In Vitro Magnetic Hyperthermia

**Figure 4:**
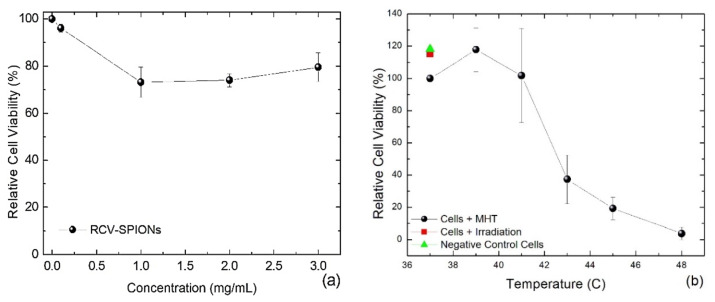
(**a**) RCV of SW620-GFP cells after 24 h of incubation with different concentrations of SPIONs and (**b**) RCV of SW620-GFP cells exposed to 2 mg/mL and heated by magnetic field irradiation from 39 up to 48 °C.

As expected, after exposure to a low concentration of 0.1 mg/mL SPIONs for 24 h, the relative cell viability (RCV) of SW620-GPF cells was not significantly affected compared to the growth control. However, even after incubation with a high concentration of 1–3 mg/mL SPIONs, the RCV was only reduced to an average of 73–85% RCV (Figure 4a). This documents the low cytotoxic effect of these SPIONs and their potential suitability for MH.

For MH, the SW620-GFP cells were incubated with 2 mg/mL SPIONs for 2 h prior to the magnetic irradiation to enhance their internalization. Then, an AC magnetic field with 530 kHz was applied, and the amplitudes were adjusted manually to reach the desired temperatures between 39 and 48 °C for 20 min. A final temperature of ≤41 °C had nearly no negative impact on the RCV; however, at 43 °C, RCV diminished drastically to <40% (Figure 4b). A further increase in temperature decreased the RCV even more, and at 48 °C, RCV was almost completely abolished, with only 4%. Additionally, the outputs represented with square and triangle are the RCV of the growth control and cells without SPIONs irradiated at 25 mT, respectively. No significant differences were observed in the RCV of both controls, because the magnetic field does not interact with cells free of SPIONs.

**Figure 5:**
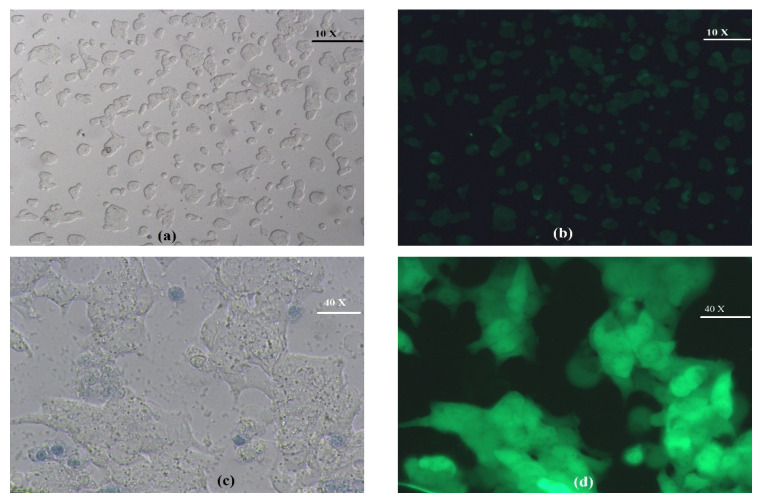

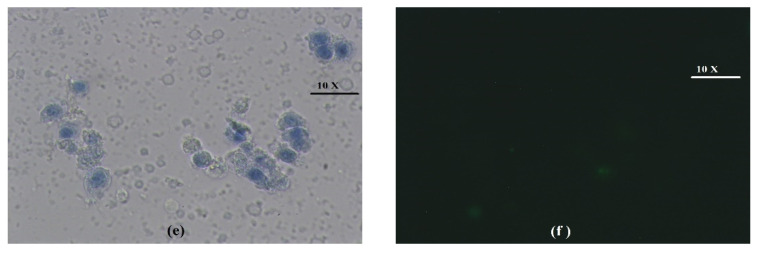
(**a**) Image of the mock control of SW620-GFP cells exposed to SPIONs but kept at 37 °C, and cells heated to 43 °C (**b**) and 48 °C (**c**) by magnetic field irradiation. (**d**–**f**) The corresponding images obtained using an epifluorescence microscope with a green filter to observe the expression of GFP.

Independently of the quantification of the RCV using a tetrazolium stain, the viability of the cells was observed and documented using trypan blue staining and epifluorescence microscopy. In the mock control, i.e., cells with SPIONs, but without heating, grew to a good confluence, and no cells were stained blue, indicating no necrotic cell death (Figure 5a). In the same frame, all non-stained cells exhibited green fluorescence, due to the expression of the GFP (Figure 5d). When the cells with SPIONs were heated to 43 °C by MH, the confluence was reduced and some blue-stained cells could be observed (Figure 5b). These blue-stained cells were not fluorescent (Figure 5e). Under the same conditions, RCV decreased to <40% (Figure 4b); now, the relatively low number of blue-stained cells in Figure 5b can be attributed to the washing process to eliminate the SPIONs, which also removed most of the dead cells. When the cells were heated to 48 °C by MH, the confluence was strongly reduced, and almost all remaining cells were stained by trypan blue (Figure 5c) and none of the stained cells exhibited green fluorescence (Figure 5f). These results confirm the cytotoxic effect of short-term MH when reaching final temperatures of 43 °C and beyond.

### 3.3. Results of In Vivo Magnetic Hyperthermia

**Figure 6:**
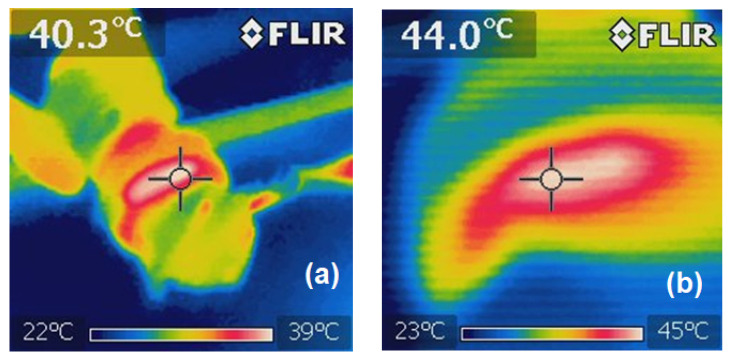
Infrared images during the irradiation procedure: (**a**) after 4 min and (**b**) after 16 min of irradiation.

During the magnetic field irradiation of the mice from group 1, the induced temperature was continuously monitored and limited to 45 °C to prevent an overheating of tissues and possible suffering of the rodents. By applying MH, the desired temperatures within the tumours could be achieved: Within 4 min of irradiation, the temperature increased to 40.3 °C (Figure 6a) and was kept at 44 °C on average (Figure 6b). It should be noted that the heating was mainly focused on the region where the SPIONs were located.

**Figure 7:**
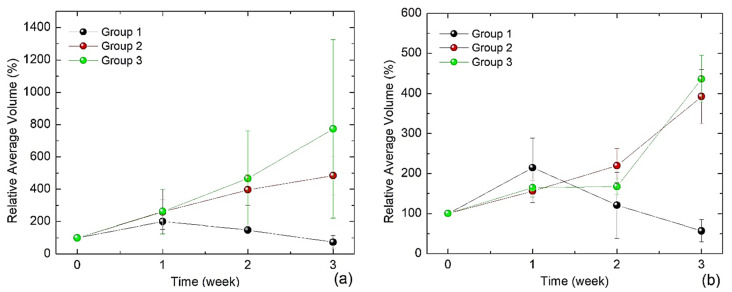
Relative average volumes (RAVs) of the tumours over time and different treatments (1 SPIONs + MH; 2 SPIONs; 3. neg. control), using (**a**) the calliper and (**b**) iBox technology. Error bars indicate standard deviation (SD).

Figure 7 summarizes the results of the evolution of the relative average volumes (RAVs) of the tumours. Only the RAV from the mice of group 1 significantly decreased after 3 weeks of treatment with MH (Figure 7, black line). When SPIONs were injected into the tumour but no MH treatment was applied (group 2), then the RAV increased continuously (Figure 7, black line) with the same growth rate as in the negative control (group 3), i.e., without any treatment (Figure 7, green line). There was no significant difference in the tumour development between groups 2 and 3 as evidenced by their overlapping error bars (SD). These results demonstrate that a direct injection of SPIONs into a subcutaneous solid tumour can successfully reduce its size, when combined with MH treatments three times a week. The RAV was measured using both, a calliper (Figure 7a) and the iBox technology (Figure 7b). Using callipers to measure tumour size is less precise, because the skin of the rodent interferes, which finally leads to larger error bars compared to the iBox technology.

**Figure 8:**
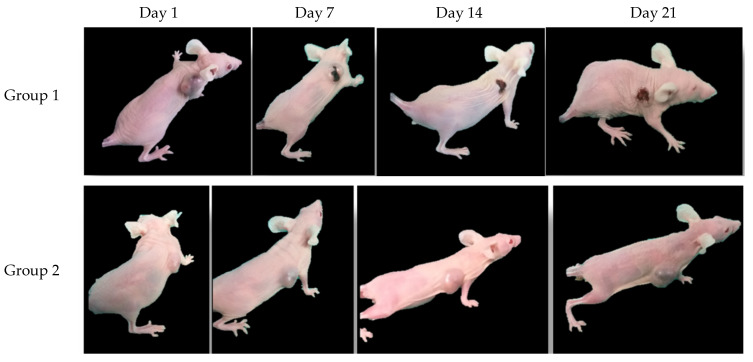

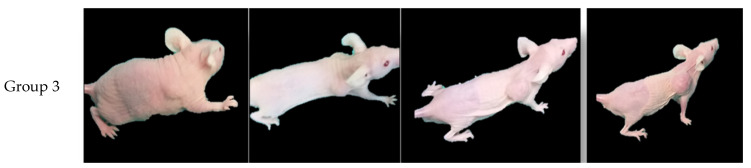
The sequence of images of one mouse from each group (1 SPIONs + MH; 2 SPIONs; 3. neg. control) at the beginning and after every seventh day. The evolution of the tumour sizes over time can be visually followed.

Additionally, the tumour development for each group was documented photographically (Figure 8): In group 1, 14 days after the injection of the tumour cells, a pronounced tumour had grown on the right shoulder of the mouse (Figure 8, group 1, day 1). However, already after two MH treatments, on day 7, the tumour size seemed to have reduced and a scab had formed, covering the wound of the remaining tumour (Figure 8, group 1, day 7). Continuing the treatment, the tumour size was further reduced, although the scab did not disappear (Figure 8, group 1, days 14 and 21). Contrary to that was the tumour development for the mice in groups 2 and 3: In both groups, the tumour size increased visually over time (Figure 8, groups 2 and 3). This visual analysis is consistent with the trends of the RAV shown in Figure 7 and discussed above.

**Figure 9:**
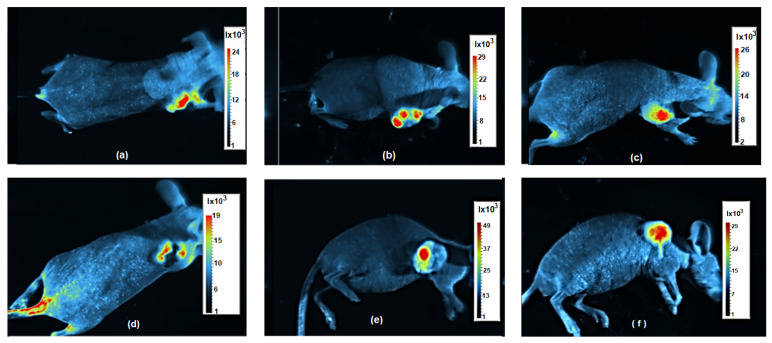
iBox images from mice after 2 weeks of MH treatment: Top view of mouse from group 1 (**a**), group 2 (**b**), and group 3 (**c**), as well as lateral view of the same mice: group 1 (**d**), group 2 (**e**), and group 3 (**f**).

With the iBox system, it was possible to determine the fluorescence of the tumour-generating SW620-GFP cells, allowing us to visualize the tumour development in response to the treatment. The intensity of the fluorescence, which correlates with the cell viability (Figure 5), increases from blue via green to red colour. After 2 weeks of treatment, the tumour size of the mice in group 1, i.e., with SPIONs and MH treatment (Figure 9a,d), was slightly smaller than both, one of the mice in group 2, i.e., with SPIONs but without MH treatment (Figure 9b,e), as well as one of the mice in group 3, i.e., the control group (Figure 9c,f). The iBox system can also overlay a millimetre grid on the captured images, which was used to measure the RAVs of the tumours presented in Figure 7b. Finally, artefacts such as fluorescence observed in the tail and leg in Figure 9d can be introduced due to sporadic hair growth; therefore, continuous inspections are necessary to avoid confusion with a metastatic process.

**Figure 10:**
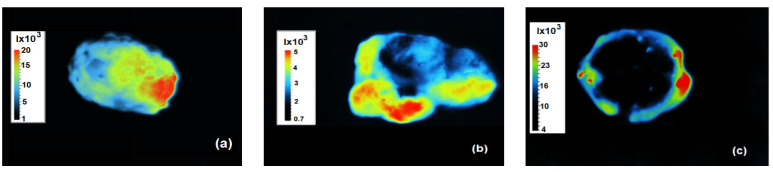
Fluorescence images of tumours extirpated from a mouse of (**a**) group 3, (**b**) group 2, and (**c**) group 1.

After 3 weeks, the mice were euthanized, and the tumours were removed to obtain a final register of their fluorescence using the iBox (Figure 10). However, some differences in the images are important to note: The tumour from a mouse in group 3 (control) was entirely fluorescent (Figure 10a). Similarly, the tumour from a mouse in group 2 exhibited fluorescence throughout all the tissue as well, although three cavities with reduced fluorescence can be observed, which were caused by a reduction in emission due to the injected SPIONs (Figure 10b). In contrast, the tumour from a mouse in group 1 showed fluorescence only in the circumference of the tissue, and the central part did not exhibit any fluorescence. This indicates the successful eradication of the tumourigenic SW620-GFP cells by MH (Figure 10c); i.e., the absence of fluorescence in the central part cannot be attributed to a reduction in fluorescence by the injected SPIONs, as it was observed in group 2.

**Figure 11:**
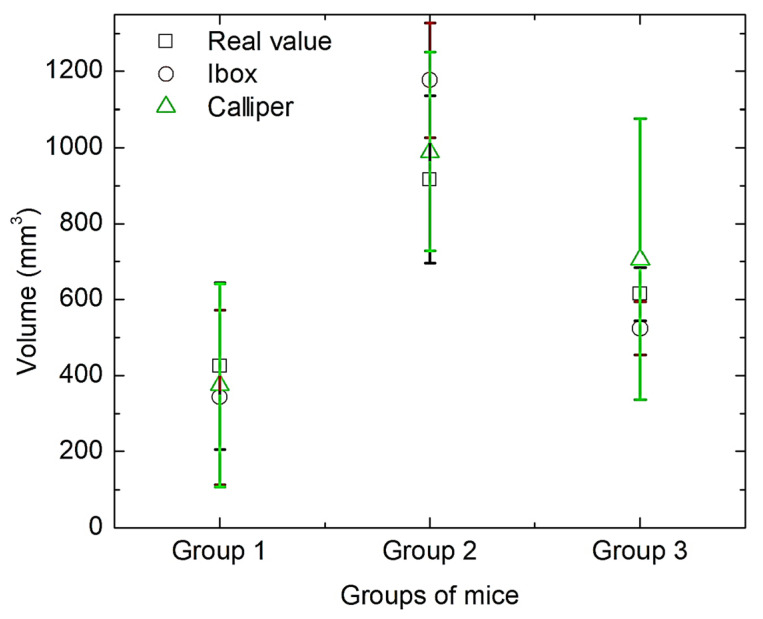
Measurements of the extirpated tumour volumes obtained by three different procedures. Error bars indicate SD.

Now, the estimated in situ volumes of the tumours, determined using calliper or iBox, after 3 weeks of treatment (Figure 7), can be compared to the real volume of the removed tumours, which was measured by weighing the mass of displaced water (Figure 11). The error bars (SD) overlapped strongly in all cases, indicating no significant differences among the different methods used to measure the volumes (at α = 0.05 level), and again, the calliper method had the highest SD.

## 4. Conclusions

This work introduces a viable alternative to carry out magnetic hyperthermia assays with fluorescent cells. One advantage over other procedures is that only viable tumoural cells exhibit fluorescence. This enables an easy and fast qualitative in vitro analysis of the cellular damage induced by heating using, e.g., an epifluorescence microscope. Additionally, the iBox technology allows for non-invasive imaging of fluorescence, similar to “radiographs”, to visualize the shape and the size of tumours at different time points during the entire magnetic hyperthermia treatment. This is also applicable for tumours growing in areas without direct access to determine their size using a calliper. Finally, in in vivo experiments, it was possible to determine non-invasively the decreasing trend of tumours after the second week of irradiation. Moreover, the final tumour sizes did not show significant differences compared to the real volume measurements.

## Data Availability

The data presented in this study are available on request from the corresponding author due to ethical reasons.
